# A meta-quantitative trait loci analysis identified consensus genomic regions and candidate genes associated with grain yield in rice

**DOI:** 10.3389/fpls.2022.1035851

**Published:** 2022-11-16

**Authors:** Kelvin Dodzi Aloryi, Nnaemeka Emmanuel Okpala, Aduragbemi Amo, Semiu Folaniyi Bello, Selorm Akaba, Xiaohai Tian

**Affiliations:** ^1^ Hubei Collaborative Innovation Centre for Grain Industry, College of Agriculture, Yangtze University, Jingzhou, China; ^2^ Institute of Plant Breeding, Genetics and Genomics University of Georgia, Athens, GA, United States; ^3^ Department of Animal Genetics, Breeding and Reproduction, College of Animal Science, South China Agricultural University, Guangzhou, Guangdong, China; ^4^ School of Agriculture, University of Cape Coast, Cape Coast, Ghana

**Keywords:** Meta-QTL analysis, rice, grain yield, Genome-Wide Association Studies, candidate genes, marker-assisted selection

## Abstract

Improving grain yield potential in rice is an important step toward addressing global food security challenges. The meta**-**QTL analysis offers stable and robust QTLs irrespective of the genetic background of mapping populations and phenotype environment and effectively narrows confidence intervals (CI) for candidate gene (CG) mining and marker-assisted selection improvement. To achieve these aims, a comprehensive bibliographic search for grain yield traits (spikelet fertility, number of grains per panicle, panicles number per plant, and 1000-grain weight) QTLs was conducted, and 462 QTLs were retrieved from 47 independent QTL research published between 2002 and 2022. QTL projection was performed using a reference map with a cumulative length of 2,945.67 cM, and MQTL analysis was conducted on 313 QTLs. Consequently, a total of 62 MQTLs were identified with reduced mean CI (up to 3.40 fold) compared to the mean CI of original QTLs. However, 10 of these MQTLs harbored at least six of the initial QTLs from diverse genetic backgrounds and environments and were considered the most stable and robust MQTLs. Also, MQTLs were compared with GWAS studies and resulted in the identification of 16 common significant loci modulating the evaluated traits. Gene annotation, gene ontology (GO) enrichment, and RNA-seq analyses of chromosome regions of the stable MQTLs detected 52 potential CGs including those that have been cloned in previous studies. These genes encode proteins known to be involved in regulating grain yield including cytochrome P450, zinc fingers, MADs-box, AP2/ERF domain, F-box, ubiquitin ligase domain protein, homeobox domain, DEAD-box ATP domain, and U-box domain. This study provides the framework for molecular dissection of grain yield in rice. Moreover, the MQTLs and CGs identified could be useful for fine mapping, gene cloning, and marker-assisted selection to improve rice productivity.

## Introduction

Rice is an important cereal crop and provides over 21% of caloric intake for more than half of the global population (Zhang et al., 2014; Vishnukiran et al., 2020; Zhao et al., 2020). Rapid population growth remains a major threat to food security worldwide, thus there is a need to augment efforts to increase the yield potential of rice. Spikelet fertility, panicle number per plant, number of grains per panicle, and thousand-grain weight are key indicators of grain yield and continue to be major targets in breeding programs (Sakamoto and Matsuoka, 2008; Huang et al., 2013; Li et al., 2019). Grain yield is quantitatively inherited and controlled by multiple loci (genes) (Xing and Zhang, 2010; Bai et al., 2012); therefore, it is imperative to ascertain the genetic bases of grain yield to boost rice productivity. With the advancement of QTL mapping, breeders have been able to identify genomic regions influencing complex agronomic traits (Daware et al., 2017) and have expedited the process of improving quantitative traits through marker**-**assisted selection (Zhang et al., 2010). However, the application of QTLs by breeders in a broader spectrum has major setbacks due to the genetic background of the populations used in QTL mapping and phenotype environment (Arcade et al., 2004; Daware et al., 2017). Therefore, a comprehensive analysis of identified QTLs is required to make these QTLs applicable in rice breeding and to decipher the underlying genetic factors conferring grain yield. Essentially, QTL meta**-**analysis has been shown to be a reliable and effective instrument (Goffinet and Gerber, 2000).

An integrated meta-QTL analysis uses QTLs from independent trials regardless of their genetic backgrounds, cross types/parents, size of populations, years, and locations to identify consensus QTLs (Goffinet and Gerber, 2000; Arcade et al., 2004; Sosnowski et al., 2012). This approach has been evidenced to reliably refine QTLs location and narrow confidence intervals (CI) which enhance the precision of marker**-**assisted selection (MAS) and discovery of candidate genes (Martinez et al., 2016; Zhang et al., 2017). Furthermore, a meta-analysis of QTLs attempts to clarify if QTLs encompassing different loci from different studies are the same loci or if they represent the same position on a linkage map of species under study. To date, numerous MQTLs for important agronomic traits have been identified in different crops such as wheat (Acuña-Galindo et al., 2015; Darzi-Ramandi et al., 2017; Soriano and Alvaro, 2019; Liu et al., 2020; Saini et al., 2021; Amo and Soriano, 2022; Miao et al., 2022; Saini et al., 2022; Yang et al., 2021), soybean (Zhao-Ming et al., 2011; Hwang et al., 2016; Qin et al., 2018), maize (Semagn et al., 2013; Wang et al., 2013; Martinez et al., 2016; Chen et al., 2017; Zhao et al., 2018; Kaur et al., 2021), barley (Zhang et al., 2017; Khahani et al., 2019), peanut (Lu et al., 2018), and sorghum (Aquib and Nafis, 2021). However, reports on Meta**-**QTLs for grain yield in rice under field conditions are limited. More so, the understanding of the molecular basis of QTLs/genes controlling grain yield is insufficient. Sandhu et al. (2021) detected 76 MQTLs governing grain number per panicle, spikelet fertility, and panicle number per plant on 29 QTL studies published from 2000 to 2022. Also, Wu et al. (2016) reported ten MQTLs associated with spikelet fertility on 82 QTL studies from 1996 to 2013. In another study, Khahani et al. (2021) integrated 101 studies in rice and confined these QTLs to meta-QTLs with reduced confidence intervals for use in MAS and discovered candidate genes for future functional validation.

This study performed a comprehensive meta**-**QTL analysis examining QTLs for spikelet fertility (SF), panicle number per plant (PN), grain number per panicle (GNP), and 1000-grain weight retrieved from 47 QTL studies published between 2002 and 2022 in 101 biparental populations evaluated across diverse environments and under field condition. The main objective of this study was to conduct meta**-**QTL analysis and identify stable and robust MQTLs and candidate genes (CGs) to aid rice breeding through MAS and enhance molecular dissection of grain yield targeted at improving the yield potential of rice.

## Materials and methods

**Figure 1:**
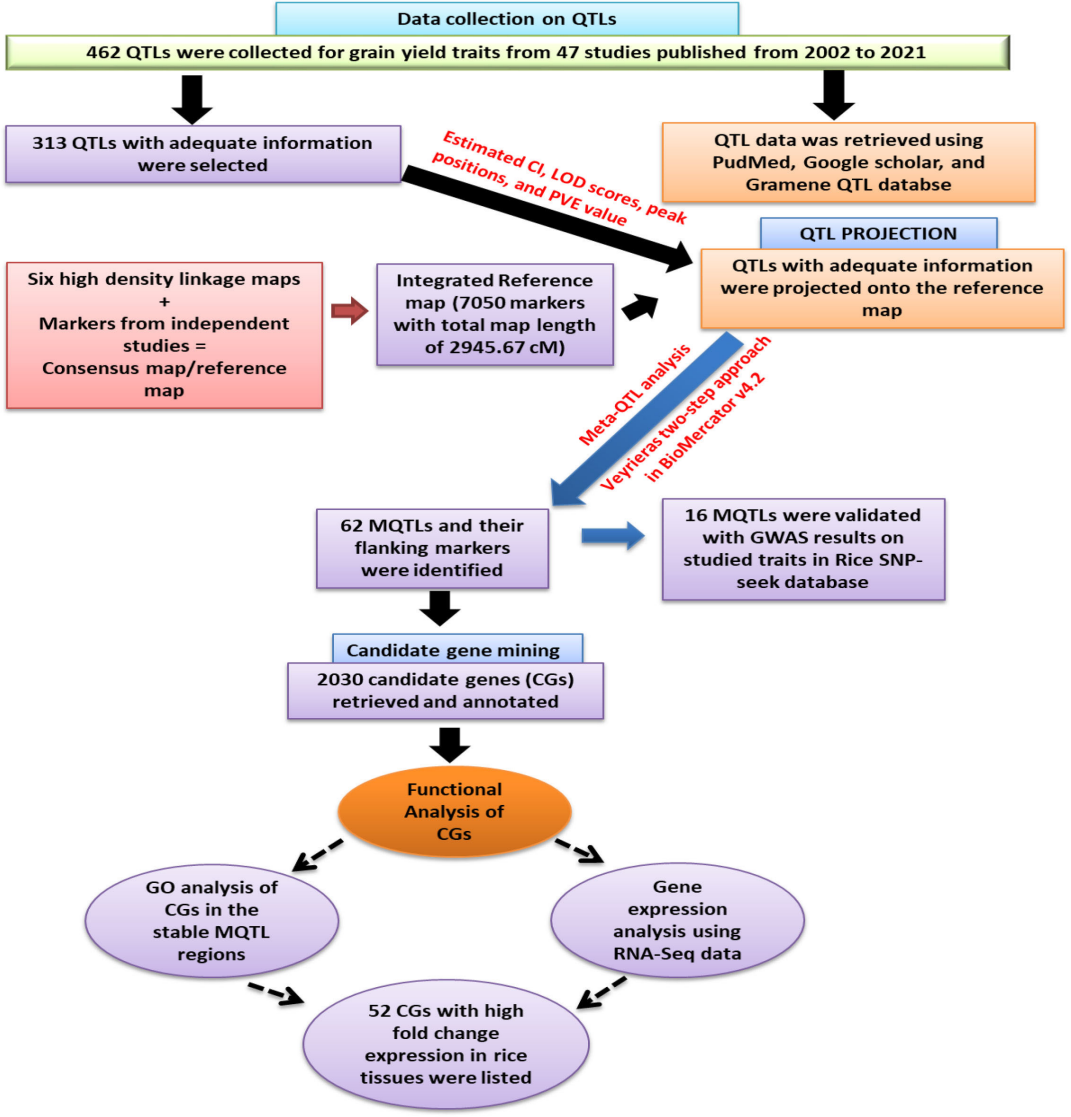
Schematic work flow and the result of the present study.

To enhance understanding, a schematic flow chart has been drawn and presented in **Figure 1**, which illustrates the approach adopted in executing this study. Further description of these steps is as ensued:

### Literature review and data collection on QTLs

A comprehensive bibliographic survey for QTLs associated with spikelet fertility (SF), grain number per panicle (GNP), number of panicles per plant (PN), and thousand-grain weight (TGW) published from 2002 to 2022 was conducted using Google Scholar (https://scholar.google.com/), PubMed (http://www.ncbi.nlm.nih.gov/pubmed), and Gramene QTL database platform (http://archive.gramene.org/qtl/) (**Table 1** and **Supplementary Table 1**). The information gathered in each study during QTL compilation was as ensued: (i) Linked markers of individual QTLs, (ii) Position (cM) of QTLs on genetic map, (iii) size and type of mapping population, (iv) phenotypic variance explained (R^2^) value of each QTLs, and (v) LOD scores of individual QTLs. The original identities of all QTLs were maintained for ease of analysis.

**Table 1:** Summary of QTL studies utilized for meta-QTL analysis.

Parents	Type of population	Size of population	No. of markers	Marker types	Traits	References
Zhenshan 97B ×Milyang 46	RIL	209	158	RFLP, SSLP	SF, PN, TGW	Zhuang et al., 2002
IR64 × Azucena	DH	135	253	RFLP, RAPDs, SSR	SF, TGW	Hittalmani et al., 2003
IR64 × IRGC 105491	BC	400	165	SSR, RFLPs	SF,PN,GNP	Septiningsih et al., 2003
IRGC 105491 × Jefersson	BC	353	153	SSR, RFLPs	SF,PN,GNP	Thomson et al., 2003
58025A x IC22015	BC	251	80	SSR	SF, PN, GN	Marri et al., 2005
Milyang 23 × Akihikari	RIL	155	273	SSR, RFLPs	GNP	Takai et al., 2005
Minghui 63 ×B5	RIL	187	244	SSR, RFLPs	PN, GNP	You et al., 2006
T226 x T219	RIL	202	181	SSR	SF	Qing-Quan et al., 2007
Suweon365 × Chucheongbyeo	RIL	231	221	SSR, ALFPs, MITEs	PN, GNP	Kwon et al., 2008
Junambyeo ×IR71033	F2	146	338	STS	SF, PN	Rahman et al., 2008
lpumbyeo (IP) × Dasanbyeo (DS)	BC	252	196	STS	SF	Qiao et al., 2008
Tarommahalli × Khazar	F2	192	74	SSR	GNP	Sabouri et al., 2009
Koshihikari × Kasalath	BC	182	162	RFLPs	TGW	Li-jun et al., 2009
Minghui 63 ×Teqing	RIL	190	133	SSR	TGW	Liu et al., 2009
Gharib × Sepidroud	F2	236	155	SSR	PN, TGW	Sabouri et al., 2010
YJCW x 93-11	BC	354	187	SSR	SF,PN,GNP, TGW	Fu et al., 2010
Zhong 156 × Gumei 2	RIL	276	198	SSR	PN,GNP,SF, TGW	Cao et al., 2010
Pusa 1266 × Pusa Basmati 1	RIL	161	175	SSR	GNP, PN	Deshmukh et al., 2010
Dasanbyeo × TR 22183	BC	166	218	SSR, STS	SF	Chin et al., 2011
Nanyangzhan × Chuan7	RIL	185	164	SSR	TGW	Bai et al., 2011
Pusa1266 x Jaya	RIL	310	162	SSR	SF,PN,GNP	Marathi et al., 2012
XieqingzaoB × Zhonghui9308	RIL	266	177	SSR	SF,PN,TGW	Liang et al., 2012
cv. D50 × cv. HB277	RIL	116	102	SSR	TGW	Tang et al., 2013
Guanghui 116 × LaGrue	RIL	307	119	SSR	GNP, TGW, SF	Zhou et al., 2013
Swarna × IRGC81848	BC	472	175	SSR	TGW	Swamy et al., 2014
HWC-line × Dasan × Hwacheong	F2	190	157	SSR, STS, FNP	SF, TGW	Seo et al., 2014
Nipponbare × 93-11	RIL	266	131	SSR	GNP, TGW	Venu et al., 2014
HP × Nipponbare	F2	93	90	SSR, InDels	GNP	Tian et al., 2015
M201 × JY293	RIL	234	32	SSR, SLAF	TGW	Xu et al., 2015
Xieqingzao B ×Zhonghui 9308	RIL	138	198	SSR	SF, PN, TGW	Yue et al., 2015
N040212 (indica) × Nipponbare	BC	146	34	SSR	SF	Ahmed et al., 2016
NYZ x Ce253	F2	188	110	SSR, InDels	TGW	Wei et al., 2016
G46B x K1075	RIL	182	4.92	SSR	TGW	Gao et al., 2016
HR1128 × Nipponbare	BC	331	37	SSR, InDels	GNP	Sun et al., 2017
R998×Francis	RIL	213	3016	SNP	PN, GNP, TGW	Zhu et al., 2017
Dasanbyeo × TR22183	RIL	179	162	SSR, STS	SF, PN, TGW	Kim et al., 2017
9IL188 x 9311	F2	152	326	SSR	GNP	Hu et al., 2018
Nagdong × Cheongcheong	RIL	90	154	SSR	PN,GNP	Jia et al., 2019
Teqing x IRBB	RIL	250	68	SSR, InDels	TGW	Zhang et al., 2020
Xuishui09 × IR2061	BC	459	6181	SNP	TGW	Wang et al., 2020
K1561 x G1025	RIL	201	5826	SSR, SLAF	TGW	Li et al., 2020
IR58025A×KMR-3R	RIL	24	1,082	SNP	TGW	Kulkarni et al., 2020
Teqing × IRBB lines, Zhenshan 97 × Milyang 46, Xieqingzao/Milyang 46	RIL	446	654	SSR	SF, GNP	Niu et al., 2020
W303 × Nipponbare	F2	166	185	SSR	TGW	Feng et al., 2021
Huanghuazhan × Jizi 1560	RIL	208	208	SSR, InDels	SF,PN, GNP	Li et al., 2021
PDK Shriram × Heera	RIL	188	133	SSR, SNP	SF	Sekhar et al., 2021
166s × 14s	F2	174	79	SSR	GNP, TGW	Beerelli et al., 2022

RIL, Recombinant inbred line; DH, Double haploid; BC, Backcross population; F_2_, Second generation of recombination. SF; spikelet fertility, PN; panicles per plant, GNP; number of grains per panicle, TGW; thousand-grain weight.

### Consensus map construction and QTL projection

The LPMerge in R package programming was used to build the reference map (Endelman and Plomion, 2014). The most comprehensive and high-density genetic map with different marker types developed by Wu et al. (2016) was selected. Further, some of the markers flanking the QTLs reported in individual studies were also incorporated into the reference map. The marker names were checked to ensure consistency and avoid duplication on the consensus map. The initial QTLs were projected using BioMercator v4.2.3 software (Sosnowski et al., 2012) (https://versailles.inra.fr/Tools/BioMercator-v4) based on the approach described in (Chardon et al., 2004). Before QTL projection, 95% confidence interval (CI) was determined using the following formulas:


$$CI=\left(\frac{530}{N\;*\;R^{2}}\right)$$

$$CI=\left(\frac{287}{N\;*\;R^{2}}\right)$$

$$CI=\left(\frac{163}{N\;*\;R^{2}}\right)$$

for backcross (BC) and F_2_, double haploid (DH), and recombinant inbred line (RIL), where N represents the size of the mapping population and R^2^ is the phenotypic variance explained by individual QTLs (Darvasi and Soller, 1997; Guo et al., 2006). 3% and 10% were used for QTLs that lack LOD scores and R^2^ values. All QTLs with adequate information required by the software were projected onto the consensus/reference map.

### Analysis of meta-quantitative trait loci (MQTLs)

Analysis of Meta-QTL was performed using BioMercator v4.2.3 (Sosnowski et al., 2012). For analysis, two approaches were used, considering the amount of QTLs in each chromosome. When the number of QTLs on each chromosome is ≤ 10, the method reported by Goffinet and Gerber (2000) was used; if the number of QTLs on each chromosome is greater than 10, the method proposed by Veyrieras et al. (2007) was utilized. Firstly, the lowest Akaike information criterion (AIC) value was selected as the best fit model. Secondly, the best fit model was considered significant in identifying MQTL from the following: AIC3, AIC, Bayesian information criterion (BIC), average weight of evidence (AWE), and corrected AIC. Based on the five criteria, the lowest value was selected to obtain MQTLs with their respective genetic positions (cM), confidence intervals (CI), and percent membership of initial QTLs to each MQTL. Using Rice SNP-seek database, all MQTLs identified in this study were verified with Genome-wide Association study results related to the studied traits (Mansueto et al., 2017). The naming of MQTL was based on their positions on the chromosome (i.e. MQTL1.1, MQTL1.2, and MQTL1.3).

### Candidate Gene (CG) excavation within the most stable MQTLs interval

To explore functional genes modulating the evaluated traits, the flanking marker sequences were employed to determine the genomic positions of MQTLs in this study. Essentially, the MQTLs with at least 6 of the initial QTLs clusters were selected as the most robust and stable MQTLs. The Gramene database (https://archive.gramene.org/markers/) and available literature were used to retrieve flanking marker sequences of the robust MQTLs. Phytozome v13 was used to blast the sequences against the rice reference genome version 7.0 (Oryza sativa v7.0) to obtain the physical positions of stable MQTLs. All model genes were extracted from the physical intervals of the stable and robust MQTLs using the rice genome annotation project database (https://rice.uga.edu/cgi-bin/gbrowse/rice/) and annotated using (http://www.ricedata.cn, https://rice.uga.edu/cgi-bin/gbrowse/rice/, http://shigen.nig.ac/jp/rice/oryzabase/, and https://rapdb.dna.affrc.go.jp/).

### Functional analysis of candidate genes

GENEDENOVO cloud platform (https://www.omicshare.com/tools/) was used to perform gene ontology (GO) enrichment analysis. The protein-protein interaction network was predicted using STRING (Szklarczyk et al., 2019). Based on the annotation information of significant CGs enriched by Gene ontology (GO) analysis, the CGs in each MQTLs with known proteins associated with grain yield were selected. In silico expression analysis was performed for these CGs using RNA-Seq gene expression data (Kawahara et al., 2013). A heatmap was constructed to visualize the expression of CGs using Morpheus online tool (https://software.broadinstitute.org/morpheus/).

## Results

### Major features of QTLs used in the meta-analysis

**Figure 2:**
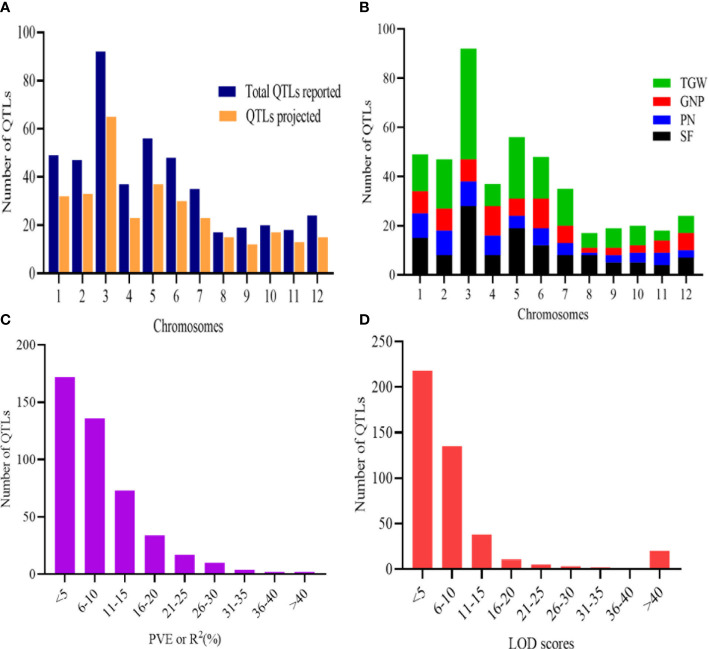
Basic features of the QTLs. **(A)** Distribution of initial QTLs and the QTLs projected. **(B)** Trait-wise distribution of QTLs on the 12 chromosomes of rice. **(C)** PVE or R2 (%) of initial QTLs. **(D)** LOD scores of initial QTL involved in MQTLs.

To unravel consensus chromosomal regions related to rice grain yield, we compiled QTL information on 462 QTLs obtained from 47 independent QTL studies (**Table 1** and **Supplementary Table 1**). The population type consisted of RIL (26), BC (11), F2 (9), and DH (1) with the mapping population size ranging from 24 to 472 (**Table 1**). The 462 QTLs were unequally distributed on all chromosomes (**Figure 2A**). A total of 197 QTLs were found on chromosomes 1, 3, and 5, followed by 47 QTLs on chromosome 2 and 48 QTLs on chromosome 6. The least QTLs (17 QTLs) were concentrated on Chr 8. Among the studied traits reported, TGW had the greatest number of QTL (179 QTL) followed by SF (127 QTL), GNP (85), and PN (71) (**Figure 2B**). Notably, most of the traits were enclosed in Chr 3, Chr 5, and Chr 6 (**Figure 2B**). QTLs explained between 0.03% and 52.0% of the phenotypic variance (PVE or R2), with an average of 8.52%. In total, 67.53% of the initial QTLs showed PVE<10%, indicating that individual QTLs explained only a small proportion of phenotype variation (**Figure 2C**). This result indicates that grain yield appears to be mostly controlled by loci with minor genetic effects and complex genetic architecture. For each QTL, LOD score ranged from 0.00 to 210.50, with an average of 10.09. The distribution of LOD scores of individual QTLs is presented in **Figure 2D**.

### Consensus map of rice

**Figure 3:**
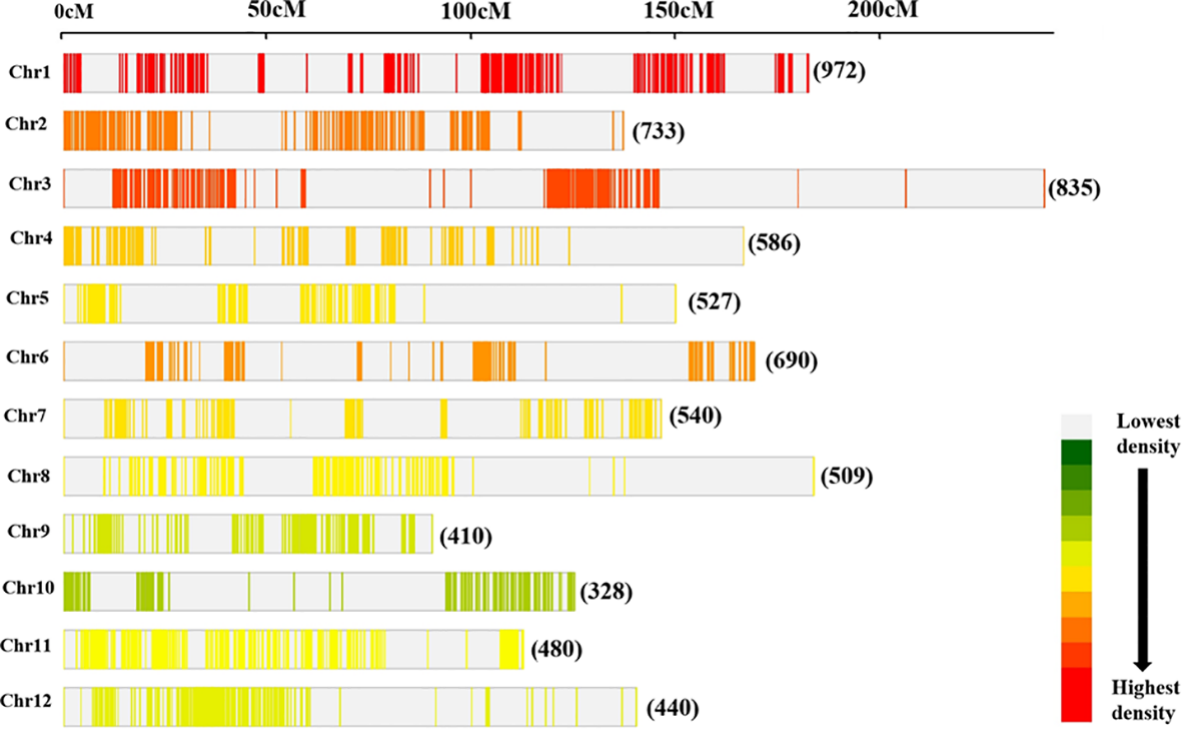
Distribution of markers on the consensus map utilized for meta-analysis of QTLs. The number of loci mapped on individual rice chromosome is shown.

The consensus map consisted of 7,050 markers (**Figure 3**) which comprised SNP, SSR, RFLPs, STS, RAPDs, ALFPs, MITEs, FNP, SLAF, SSLP, and InDels. Also, genes such as *Grh*, *Prp2*, *Pox5*, *Pla1*, *Rpr2*, *W1*, and *cen1* were included in the reference map. The consensus/reference map had a cumulative map length of 2,945.67 cM with a genetic distance of individual chromosomes ranging from 144.00 cM to 383.80 cM (**Supplementary Table 2**). The mean marker densities per chromosome ranged from 7.25 cM to 21.49 cM and 13.10 cM for the whole genome. The highest number of markers were found on Chr 1 (972 markers) followed by Chr 3 (835 markers) and Chr 2 (733 markers) (**Figure 3**). However, Chr 10 harbored the lowest number of markers (328 markers) on the reference map.

### QTL projection and analysis of meta-QTL

**Figure 4:**
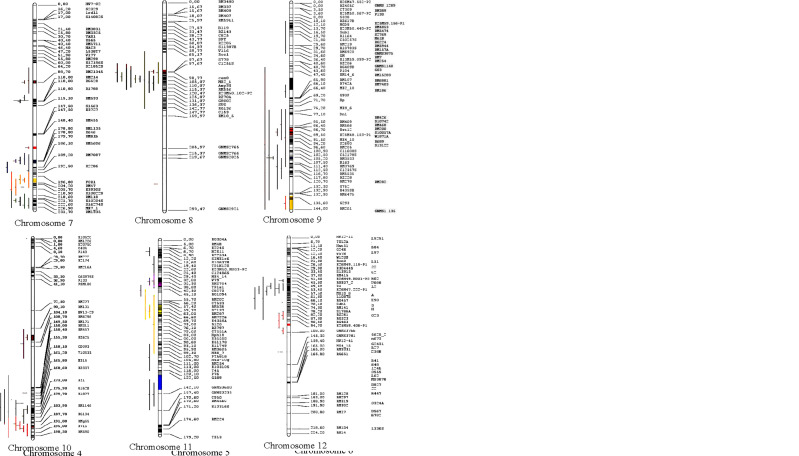
Distribution of MQTLs for grain yield traits on the 12 chromosomes of rice. Different colors on the left side of the maps indicate the initial QTLs involved in MQTLs.

Of the total 462 QTLs available, only 313 QTLs could be successfully projected onto the reference map; the remaining 149 QTLs could not be projected due to inadequate information required for the QTL projection. The MQTL analysis refined the projected QTLs (313 QTLs) to 62 MQTLs (**Figure 4** and **Table 2**). There was an uneven distribution of MQTLs on the rice chromosome. MQTLs on each chromosome ranged from two (Chr 8) to nine (Chr 3) with an average of 5.16 MQTLs per chromosome. The projection yielded the detection of 62 MQTLs, which involved 232 QTLs (**Table 2** and **Figure 4**); the remaining 81 QTLs could not be allocated to any of the MQTLs partly due to lack of associated markers on the reference map and initial maps, low phenotypic variance explained by individual QTLs, relatively low LOD score values, and large confidence interval (CI), respectively.

**Table 2:** Summary of meta-QTLs identified in this study.

MQTLs	Chr	Position (cM)	Flanking markers	No. of QTLs	No. of Trait	Traits (avg. PVE)^a^
MQTL1.1	1	46.48	RM6324-C11461	8	3	SF,GNP,TGW (11.09)
MQTL1.2	1	62.9	GNMS3879-RM583	1	1	GNP (27.6)
MQTL1.3	1	84.25	RM600-RM572	2	2	SF,TGW (4.94)
MQTL1.4	1	116.8	RM23-RM24	1	1	SF (4.8)
MQTL1.5	1	176.13	TP1E3B-RM3143	4	2	GNP,TGW (9.03)
MQTL1.6	1	199.86	C10980S-C1162	3	3	SF,TGW (16.05)
MQTL1.7	1	224	C1162-RM212	4	2	SF,TGW (5.81)
MQTL1.8	1	257.89	G54-RM5310	1	1	SF (0.09)
MQTL2.1	2	2.19	C60318SC-RRH02_2	1	1	GNP (18.22)
MQTL2.2	2	27.14	RM6911-RM324	5	2	PN,TGW (7.85)
MQTL2.3	2	65.83	S20660A-RG157	3	1	TGW (8.53)
MQTL2.4	2	118.36	C920-L107	3	1	SF (4.73)
MQTL2.5	2	145.51	Rf2-RM240	4	3	SF,GNP,TGW (11.35)
MQTL2.6	2	161.74	Y8007R-RM208	7	3	SF,GNP,TGW (7.87)
MQTL3.1	3	57.62	RM5474-GNMS3875	4	3	SF,GNP,TGW (0.86)
MQTL3.2	3	115.3	RM254-GNMS1140	4	2	SF,TGW (12.8)
MQTL3.3	3	153.71	GS3-RM15283	3	2	SF,TGW (11.38)
MQTL3.4	3	191.94	RM6681-RM426	4	1	TGW (17.11)
MQTL3.5	3	197.49	RM426-S10742	14	4	SF,GNP,PN,TGW (10.52
MQTL3.6	3	205.15	S10742-RM468	2	1	TGW (23.7)
MQTL3.7	3	224.72	RM200-S10057A	4	2	SF,TGW (8.41)
MQTL3.8	3	253.74	W1871A-R689	5	2	SF,TGW (6.9)
MQTL3.9	3	258.92	R689-S13122	5	3	SF,GNP,TGW (12.13)
MQTL4.1	4	30.25	RM6314-RM2536	2	1	SF (11.4)
MQTL4.2	4	130.54	RM273-RG776A	7	3	SF,GNP,TGW (8.89)
MQTL4.3	4	143.05	RG776A-Prp2	2	1	GNP (11.13)
MQTL4.4	4	160.52	PSM115-RM5709	1	1	SF (0.19)
MQTL4.5	4	172.31	RM7509-C1016	5	2	GNP,TGW (4.98)
MQTL5.1	5	16.88	Rpr2-RM153	2	1	SF (7.79)
MQTL5.2	5	64.27	E61293S-Pi23	13	3	SF,GNP,TGW (11.77)
MQTL5.3	5	67.95	Pi23-RM509	4	3	SF,GNP,TGW (10.00)
MQTL5.4	5	81.44	RM161-RM305	1	1	TGW (18.13)
MQTL5.5	5	104.18	RM3351-RZ225	6	3	SF,GNP,TGW (9.14)
MQTL6.1	6	69.42	RM7088-TaA	4	2	SF,GNP (10.35)
MQTL6.2	6	89.51	RM190-VB3	2	2	SF,TGW (2.8)
MQTL6.3	6	116.23	LDH-G8023	6	3	SF,GNP,TGW (8.16)
MQTL6.4	6	152.51	RM238B-C1	1	1	SF (0.25)
MQTL6.5	6	162.41	RG468-Y1124L	3	3	SF,GNP,TGW (7.97)
MQTL6.6	6	229.01	GNMS3878-RM3827	2	1	GNP (6.9)
MQTL6.7	6	270.28	RM4447-E31330S	4	2	SF,TGW (6.14)
MQTL7.1	7	88.77	RM21345-RM215	2	1	TGW (10.46)
MQTL7.2	7	109.5	RM214-R1788	3	2	SF,GNP (9.34)
MQTL7.3	7	164.15	RM455-RM1135	1	1	SF (0.19)
MQTL7.4	7	179.68	RM1135-R2286	5	3	SF,GNP,TGW (8.61)
MQTL7.5	7	201.31	FOR1-R10022S	4	2	SF,GNP,TGW (4.43)
MQTL7.6	7	225.17	S162745-RM1335	4	2	GNP,TGW (9.42)
MQTL8.1	8	97.84	S779-C12515	1	1	SF (15.3)
MQTL8.2	8	107.56	cen8-RM556	10	3	SF,GNP,TGW (6.64)
MQTL9.1	9	56.41	EM14_6-RM107	1	1	SF (4.0)
MQTL9.2	9	89.89	RM566-ME4_10	4	4	SF,GNP,PN,TGW (6.51)
MQTL9.3	9	108.16	B163-RM3769	5	3	SF,GNP,PN (3.59)
MQTL9.4	9	139.64	G293-RM201	1	1	TGW (8.07)
MQTL10.1	10	100.03	RM101-RM2696	2	2	GNP,TGW (16.28)
MQTL10.2	10	173.08	RZ337-R1877	6	2	SF,TGW (7.58)
MQTL10.3	10	190.05	RG134-RMg65	4	2	SF,TGW (16.2)
MQTL11.1	11	13.15	SINE1r6-S10637B	2	2	SF,GNP (14.95)
MQTL11.2	11	40.39	TP1s1-RG1094	3	2	SF,TGW (7.52)
MQTL11.3	11	65.37	S2137-E435sA	3	2	GNP,PN (4.4)
MQTL11.4	11	131.53	G389-GNMS3600	2	2	GNP,PN (5.75)
MQTL12.1	12	49.27	P4-E26M47.222-P1	1	1	TGW (24.81)
MQTL12.2	12	58.07	RG457-Sdh1	8	2	SF,GNP (7.81)
MQTL12.3	12	99.68	RG323-GNMS3766	3	2	SF,GNP.TGW (4.02)

a Average phenotypic variance explained.

**Figure 5:**
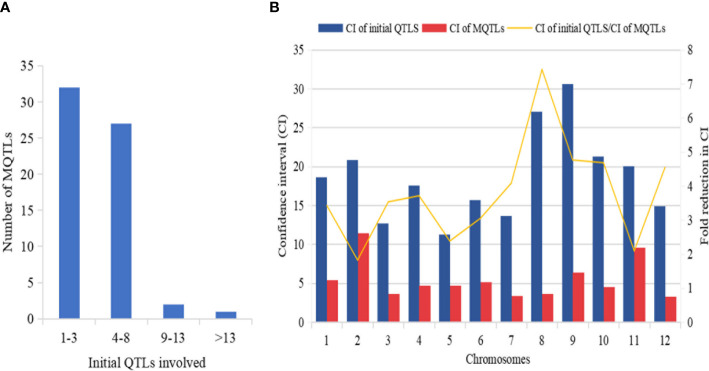
Basic information of MQTLs identified in this study. **(A)** Frequency of MQTLs and the number of initial QTLs involved. **(B)** Comparison of CI of initial QTLs and MQTLs, exhibiting fold reduction in CI of initial QTLs and MQTLs.

The explained phenotypic variation by each MQTL identified ranged from 0.09% to 27.6% with a mean PVE (%) of 9.26% (**Table 2**). Individual MQTLs differed markedly for many traits modulating MQTL (**Table 2**). The MQTLs association with studied traits varied markedly from a solitary trait controlled by MQTL1.2 to 14 traits regulated by MQTL3.5 (**Figure 5A**). Out of 62 MQTLs, 50 MQTLs were related to at least two QTLs (controlling more than two different traits) from different mapping populations and genetic backgrounds. This result showed significant co-localization of chromosome regions regulating grain yield in rice. A total of 7 MQTLs (MQTL1.1, MQTL2.6, MQTL4.2, MQTL5.5, MQTL6.3, MQTL10.2, and MQTL12.2) were identified to contain more than six QTLs (**Table 2**). The 3 MQTLs (MQTL3.5, MQTL5.2, and MQTL8.2) were detected to contain more than 10 QTLs. However, 12 MQTLs (MQTL1.1, MQTL1.4, MQTL1.8, MQTL2.1, MQTL4.4, MQTL5.4, MQTL6.4, MQTL7.3, MQTL8.1, MQTL9.1, MQTL9.4, and MQTL12.1) were reported to harbor a single initial QTL governing spikelet fertility, thousand-grain weight, and grain number per panicle. Interestingly, none of the MQTLs showed overlapped regions in this study.

The mean confidence interval of the MQTLs showed a markedly significant reduction (3.40 fold) compared to the CI of the initial QTLs (**Figure 5B**). The CI of initial QTLs ranged from 0.60 cM to 112.29 cM with a mean CI of 18.03. The CI of the reported MQTLs ranged from 0.24 cM to 29.45 cM with a mean CI of 10.91 cM. The average CI of MQTLs per chromosome showed a narrow CI ranging from 3.29 cM to 9.58 cM compared to the CI of initial QTLs which showed a wider CI ranging from 11.32 cM to 30.59 cM (**Figure 5B**), respectively. The fold reduction (average) in the size of CI of MQTLs was highest for Chr 8 (7.42 fold) followed by Chr 9 (4.76 fold); Chr 2 (1.80 fold) recorded the lowest fold reduction in the size of CI of MQTLs (**Figure 5B**).

### Validation of MQTLs with GWAS

To check the efficacy of identified MQTLs, the physical position of all MQTLs was compared with the GWAS results available on Rice SNP-seek database. Consequently, 16 MQTLs co-located with SNP peak loci in rice GWAS for 1000-grain weight and spikelet fertility traits (**Supplementary Figure 1**). Among the 16 MQTLs, only two MQTLs (MQTL3.2 and MQTL3.5) were co-located with GWAS signals for both 1000-grain weight and spikelet fertility. This indicates the coherence of the two methods in identifying common chromosome regions associated with the studied traits.

### Candidate gene mining of the most stable and robust MQTLs

**Figure 6:**
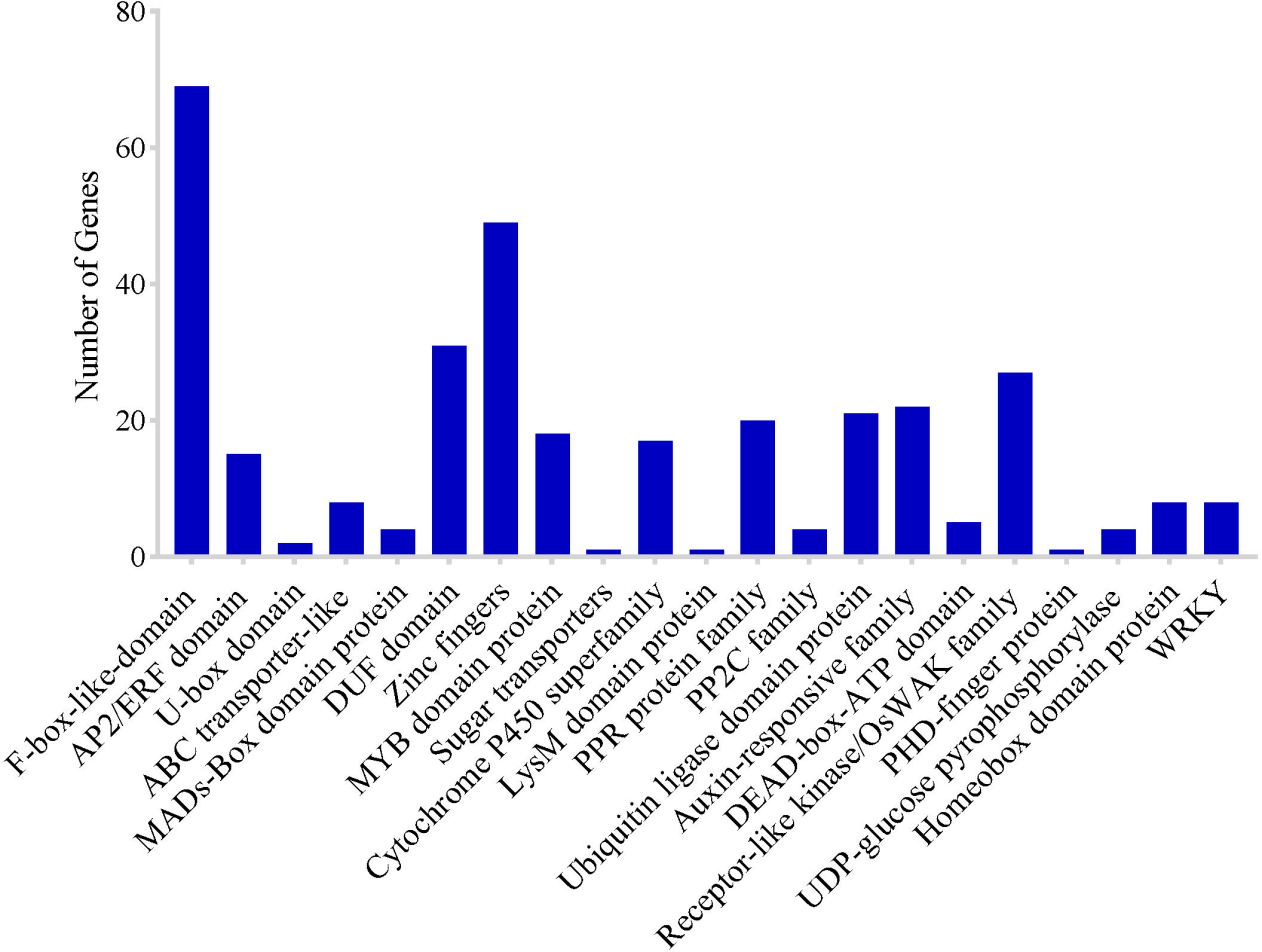
Frequency of candidate genes encoding known protein families related to grain yield of rice.

To identify potential CGs in the MQTLs regions, MQTLs that harbored at least six of the initial QTL clusters were selected as the most stable and robust MQTLs. Based on this, 10 of the 62 MQTLs were selected. However, CGs could not be extracted in the two MQTL intervals due to the unavailability of flanking marker sequences. A total of 2,030 putative CGs underlying 8 MQTLs regions were identified including 321 genes of unknown function (**Supplementary Table 3**). There were numerous genes and/or gene families with related functions in various MQTL intervals (**Figure 6**) which included: 69 F-box-like domain, 15 AP2/ERF domain, 2 U-box domain protein, 8 ABC transporter-like, 4 MADs-Box domain-containing protein, 31 DUF domain proteins, 49 zinc finger domain-containing protein, 18 MYB family protein, 1 sugar transporter, 17 cytochrome P450, 1 Lysm domain-containing protein, 20 pentatricopeptide repeat protein family, 4 protein phosphatase 2C family, 21 ubiquitin ligase domain protein, 22 auxin-response family protein, 5 DEAD-box-ATP families, 27 receptor-like kinase/OsWAK family, 1 PHD finger protein, 4 UDP-glucose pyrophosphorylase protein, and 8 homeobox domain-containing protein (**Figure 6**). These genes were chosen for more detailed study on the basis that they encoded proteins directly associated with grain yield. Clusters of genes with similar functions were also identified in some MQTL regions which included: (i) F-box protein, (ii) prolamin precursor, expressed, (iii) ubiquitin carboxyl-terminal hydrolase, family 1, (iv) retrotransposons, and (v) zinc fingers (**Supplementary Table 3**).

### Gene Ontology (GO) and expression of CGs

**Figure 7:**
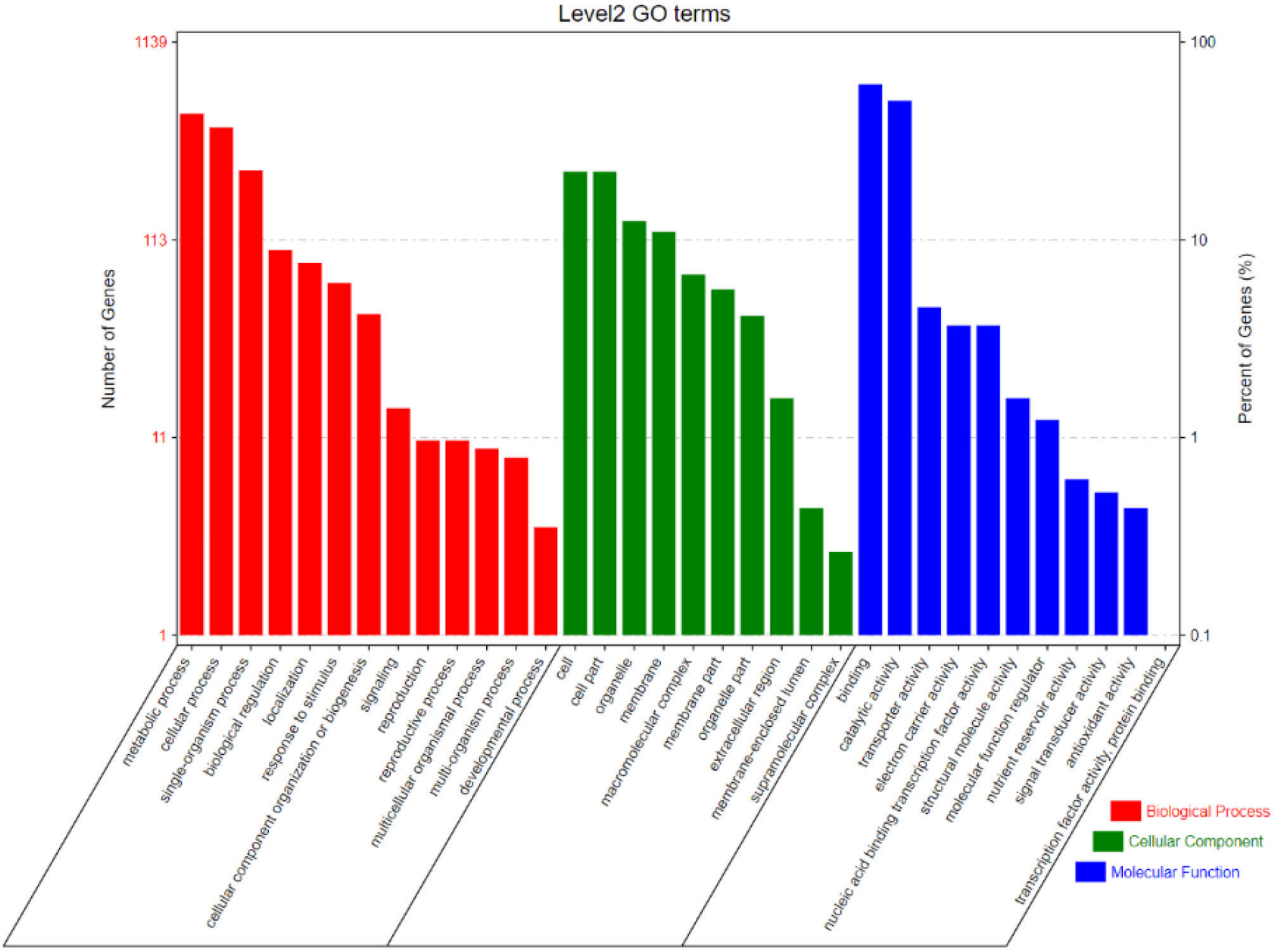
Level 2 Gene ontology (GO) terms for CGs in the most stable and robust MQTLs intervals.

**Figure 8:**
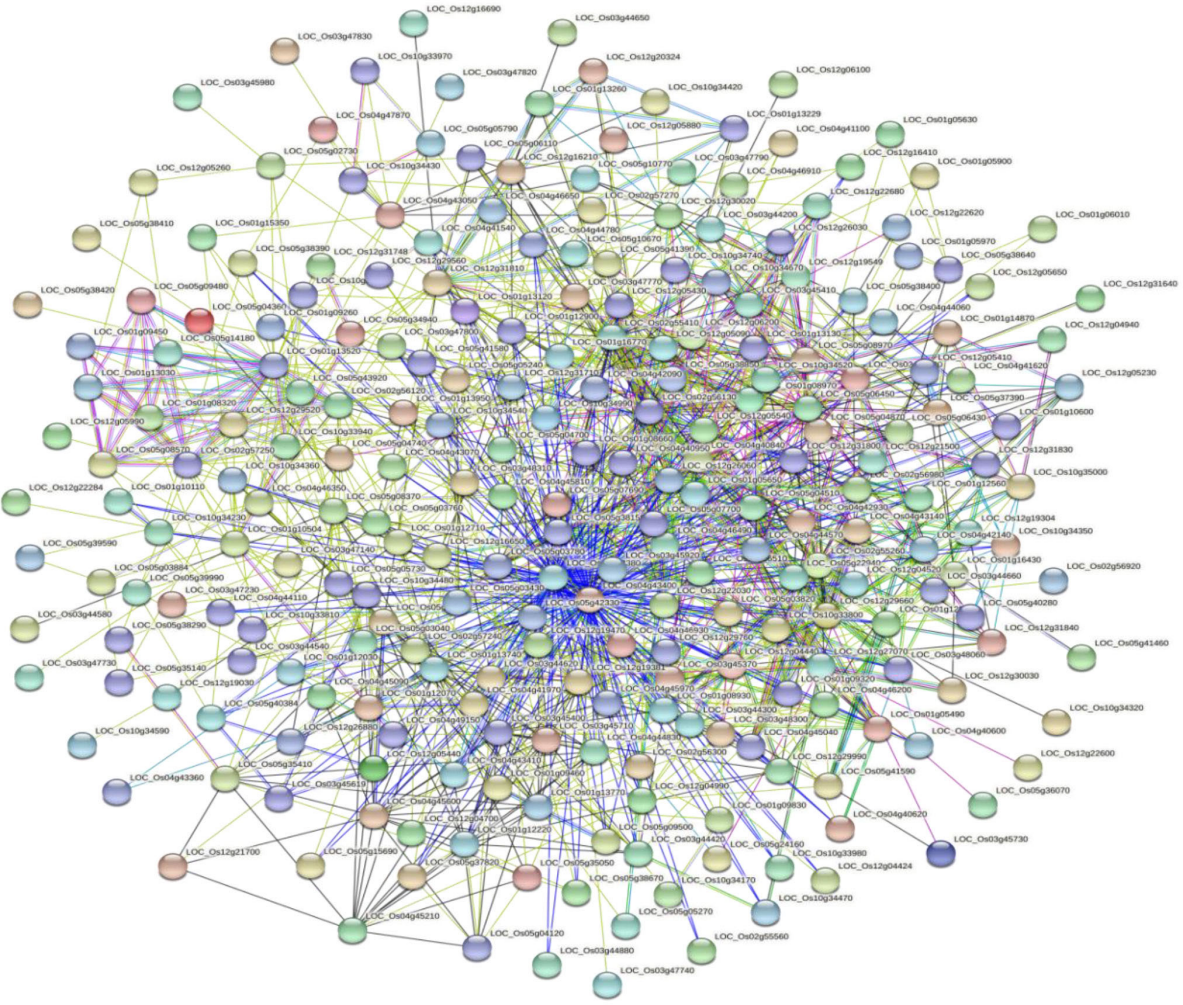
Protein-protein interaction of significant candidate genes regulating grain yield.

Based on the GO enrichment analysis, 1,139 out of 2,030 CGs were annotated of which 34 GO terms were significantly enriched (**Figure 7**). Out of the 34 GO terms, 13, 10, and 11 GO terms were significantly enriched in biological process, cellular component, and molecular function, respectively. The most enriched GO terms in the biological process category were: metabolic process, cellular process, and single-organism process. For the cellular component, the most enriched GO terms were: cell, cell part, organelle, and membrane. High percentages of genes were enriched in the molecular process category which included binding and catalytic activity (**Figure 7**). These results indicate the significant association of these terms with grain yield in rice. The CGs significantly enriched during GO analysis were further subjected to protein-protein interaction to predict their relationships. Based on the analysis, six key interactions were determined, including an interaction between *LOC_Os04g43410* and 109 genes, *LOC_Os04g43380* and 101 genes, *LOC_Os02g56130* and 43 genes, *LOC_Os01g13520* and 33 genes, *LOC_Os02g55410* and 29 genes, and *LOC_Os12g26060* and 25 genes (**Figure 8**), respectively.

**Figure 9:**
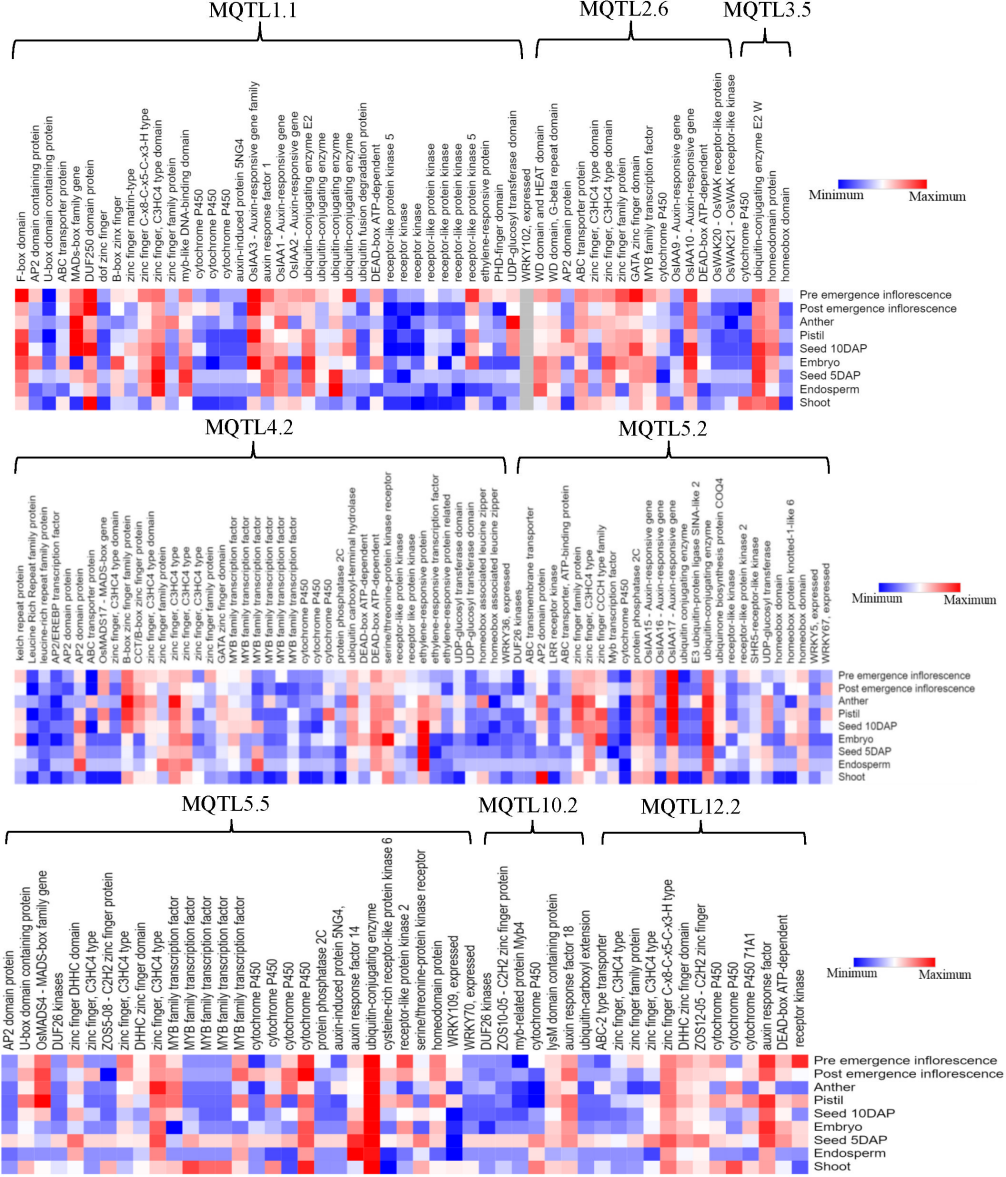
Expression profile of significant proteins encoded by 176 candidate genes underlying the following 8 MQTLs regions: MQTL1.1, MQTL2.6, MQTL3.5, MQTL4.2, MQTL5.2, MQTL5.5, MQTL10.2, and MQTL12.2.

To discover potential CGs underlying the promising MQTLs genomic regions, we focused on CGs which encode proteins associated with grain yield in rice (**Figure 6**). A total of 327 CGs were identified to belong to the above proteins. Moreover, the RNA-seq data of genes that is publicly available on the rice genome annotation project database were employed to heatmap the expression of the CGs in pre-emergence inflorescence, post-emergence inflorescence, anther, pistil, seed 10DAP, embryo, seed 5DAP, endosperm, and shoot (**Figure 9**). Based on the heatmap, 52 of the 327 genes showed high fold change expression in anther, pistil, seed, embryo, and inflorescence (**Figure 9** and **Table 3**); hence, they could be grain yield regulatory genes. The MQTL1.1 possessed the highest number of potential CGs followed by MQTL4.2, MQTL2.6, MQTL5.5, MQTL5.2, and MQTL12.2. However, MQTL3.5 and MQTL10.2 had the lowest number of genes with high expression in yield-related tissues (**Table 3**).

**Table 3:** Annotation of potential candidate genes underlying Meta-QTL regions.

MQTL	Gene ID	Description	Gene symbol
MQTL1.1	*LOC_Os01g05970*	OsFBO1-F-box and other domain containing protein	*OsFBO1*
	*LOC_Os01g12440*	AP2 domain containing protein, expressed	*ERF53*
	*LOC_Os01g10504*	OsMADS3 - MADS-box family gene with MIKCc type-box	*MADS3*
	*LOC_Os01g13770*	DUF250 domain containing protein	*OsTPT1*
	*LOC_Os01g10580*	B-box zinc finger family protein	*OsBBX1*
	*LOC_Os01g15630*	zinc finger, C3HC4 type domain	*OsRFP*
	*LOC_Os01g13740*	myb-like DNA-binding domain	*OsGLK2*
	*LOC_Os01g13030*	OsIAA3 - Auxin-responsive Aux/IAA gene family member	*OsIAA3*
	*LOC_Os01g13170*	ubiquitin-conjugating enzyme E2	*OsUBC42*
	*LOC_Os01g13280*	ubiquitin conjugating enzyme protein	*OsUBC37*
	*LOC_Os01g16650*	ubiquitin-conjugating enzyme	*OsUBC27*
	*LOC_Os01g08930*	DEAD-box ATP-dependent RNA helicase	*OsRH39*
	*LOC_Os01g05640*	receptor-like protein kinase 5 precursor	–
	*LOC_Os01g08440*	UDP-glucoronosyl and UDP-glucosyl transferase domain	*OsUGT75K1*
MQTL2.6	*LOC_Os02g55340*	WD domain and HEAT domain containing protein	*OsWD40-57*
	*LOC_Os02g55380*	AP2 domain containing protein	*OsERF127*
	*LOC_Os02g56550*	ABC transporter, ATP-binding protein	*OsABCI14*
	*LOC_Os02g55520*	zinc finger, C3HC4 type domain	–
	*LOC_Os02g56280*	zinc finger family protein, putative, expressed	–
	*LOC_Os02g56250*	GATA zinc finger domain containing protein	*OsGATA3*
	*LOC_Os02g57250*	OsIAA10 - Auxin-responsive Aux/IAA gene family member	*OsIAA10*
MQTL3.5	*LOC_Os03g47770*	ubiquitin-conjugating enzyme E2 W	*OsUBC25*
	*LOC_Os03g47740*	homeodomain protein, putative, expressed	*OsBIHD1*
MQTL4.2	*LOC_Os04g46440*	AP2 domain containing protein, expressed	*OsERF34*
	*LOC_Os04g41560*	B-box zinc finger family protein	*OsBBX11*
	*LOC_Os04g44820*	zinc finger, C3HC4 type domain	*OsRDCP1*
	*LOC_Os04g45020*	MYB family transcription factor	–
	*LOC_Os04g48460*	cytochrome P450, putative, expressed	*OsCYP704A3*
	*LOC_Os04g46190*	ubiquitin carboxyl-terminal hydrolase, family 1, putative, expressed	*OsUCH4*
	*LOC_Os04g42740*	serine/threonine-protein kinase receptor precursor, putative, expressed	–
	*LOC_Os04g41570*	ethylene-responsive protein related, putative, expressed	*OsbHLH65*
	*LOC_Os04g45810*	homeobox associated leucine zipper, putative, expressed	*Oshox22*
MQTL5.2	*LOC_Os05g03040*	AP2 domain containing protein, expressed	*RSR1*
	*LOC_Os05g03760*	zinc finger family protein, putative, expressed	*OsTZF5*
	*LOC_Os05g06270*	zinc finger, C3HC4 type domain containing protein	*OsAPIP6*
	*LOC_Os05g10670*	zinc finger CCCH type family protein, putative	*OsTZF1*
	*LOC_Os05g14180*	OsIAA17 - Auxin-responsive Aux/IAA gene family member	*OsIAA17*
	*LOC_Os05g08960*	ubiquitin-conjugating enzyme, putative	*OsUBC8*
MQTL5.5	*LOC_Os05g36360*	U-box domain containing protein, expressed	*OsPUB44*
	*LOC_Os05g34940*	OsMADS4 - MADS-box family gene with MIKCc type-box	*OsMADS4*
	*LOC_Os05g36090*	zinc finger DHHC domain-containing protein	–
	*LOC_Os05g39380*	zinc finger, C3HC4 type domain containing protein	*OsRFP*
	*LOC_Os05g41440*	cytochrome P450, putative, expressed	*CYP98A4*
	*LOC_Os05g43920*	auxin response factor 14, putative, expressed	*OsARF14*
	*LOC_Os05g38550*	ubiquitin-conjugating enzyme, putative	*OsUBC12*
MQTL10.2	*LOC_Os10g34480*	cytochrome P450, putative, expressed	–
	*LOC_Os10g33940*	auxin response factor 18, putative, expressed	*OsARF22*
MQTL12.2	*LOC_Os12g21700*	zinc finger C-x8-C-x5-C-x3-H type family protein	*OsC3H66*
	*LOC_Os12g31840*	ZOS12-05 - C2H2 zinc finger protein	*OsDjC81*
	*LOC_Os12g05440*	cytochrome P450, putative, expressed	C*YP94C2b*
	*LOC_Os12g29520*	auxin response factor, putative, expressed	*OsARF24*
	*LOC_Os12g05120*	receptor kinase, putative, expressed	–

## Discussion

Increasing the productivity of rice is one of the foremost constraints rice breeders face to ensure adequate food supply to a rapidly growing global population. Most important agronomic traits such as spikelet fertility, number of grains per panicle, panicle number per plant, and thousand-grain weight are complex and modulated by numerous polygenic loci. Over the years, the success in the introgression of QTLs in breeding programs by marker-assisted selection has been slow due to inconsistency in QTL genetic backgrounds and phenotype environment. Integration of QTLs information from diverse independent studies and exploiting meta-analysis to identify MQTL regions has proven to be an ideal approach to accelerating QTLs fine mapping and gene cloning (Wang et al., 2013; Wang et al., 2015; Martinez et al., 2016; Wu et al., 2016; Chen et al., 2017).

To decipher the molecular mechanism underlying grain yield, MQTL analysis was conducted based on the QTLs conferring grain yield collected from previous independent studies. The initial step in the meta-analysis of QTL is the QTL projection onto the reference map. A characteristics of the reference map utilized in the current study showed that the highest marker saturation was reported on chromosome 1 followed by chromosomes 3 and 2 with the lowest marker density found on chromosome 10. Individual chromosomes had uneven marker distribution, and the density of markers at either end of the chromosome differed. This is primarily due to the use of independent genetic maps with different marker types and number of markers. Interestingly, similar cases have been reported in earlier studies on meta-analysis of QTLs (Venske et al., 2019; Liu et al., 2020; Saini et al., 2021; Yang et al., 2021; Saini et al., 2022). More so, the QTLs investigated were not evenly distributed across the entire rice chromosomes. Chromosomes 1, 2, 3, and 5 reported the highest number of QTLs which is consistent with the previous studies (Xing and Zhang, 2010; Swamy and Sarla, 2011; Swamy et al., 2011; Wu et al., 2016; Khahani et al., 2020). However, chromosomes 8, 9, and 10 reported the lowest number of QTLs. The possible reason for this phenomenon could be the low level of polymorphism associated with these chromosomes. Out of the total QTLs projected, only 32.47% constitute PVE >10%, indicating that the individual QTLs contributed very little to phenotypic variability. This shows that grain yield is primarily determined by minor effects loci, representing a complex genetic architecture.

The MQTL analysis identifies stable and robust QTLs irrespective of the genetic background of mapping population, phenotype environment, and marker density which are the main constraints of QTL mapping (Arcade et al., 2004; Zhang et al., 2010; Daware et al., 2017; Bilgrami et al., 2018; Khahani et al., 2019). Further, association mapping approach has also been shown as an alternative instrument with better accuracy compared to QTL mapping of complex agronomic traits (Daware et al., 2017). However, this method faces remarkable false positive outcomes because of the population structure employed in the analysis (Daware et al., 2017). Owing to this, analysis of MQTL is regarded as the most reliable approach for detection of stable loci modulating quantitative traits. In this study, a total of 313 QTLs were confined into 62 MQTLs across the 12 chromosomes for the evaluated traits. Chromosome 3 reported the highest number of MQTLs (9 MQTLs) and the least MQTL were located on chromosome 8 (2 MQTLs). Intriguingly, our findings corroborate with a previous study on MQTLs in rice (Khahani et al., 2021). More than 80% (50/62) of MQTLs harbored at least two of the initial QTLs. Furthermore, some of the MQTLs contained as high as 10 initial QTLs exhibiting MQTLs robustness. The reference map developed and utilized in this study is much more informative compared to those employed in the previous studies (Wu et al., 2016; Khahani et al., 2020; Khahani et al., 2021). We also found that six out of the total MQTLs identified had common genomic positions with previously reported MQTLs (Wu et al., 2016; Sandhu et al., 2021). This includes regions on chromosomes 2 (MQTL2.3), 4 (MQTL4.1, MQTL4.2), 7 (MQTL7.2), 9 (MQTL9.1), and 12 (MQTL12.1). It is worth noting that, the average confidence interval of MQTLs remarkably reduced up to 3.4 folds times compared to the confidence interval of original QTLs. Our study clearly shows that the meta-analysis is effective in locating consensus and exact QTLs. Previous studies on meta-analysis in wheat, rice, maize, soybean, and cotton, reported 10% to 21% reduction in the total QTLs with two to four times reduction in the average confidence interval of original QTLs (Guo et al., 2006; Rong et al., 2007; Ballini et al., 2008; Courtois et al., 2009; Lanaud et al., 2009; Löffler, 2009; Khahani et al., 2020). MQTLs identified were compared with GWAS studies *via* the Rice SNP-seek database which led to the identification of common significant regions. Consequently, 16 (25.81%) important GWAS peaks for spikelet fertility (SF) and 1000-grain weight (TGW) were co-located with our MQTLs. Our results demonstrate the coherence of the two approaches in pinpointing common loci associated with the studied traits. However, there are also reports which contradict the result in the present study with 63%, 61.3% and 38.66% MQTLs validated with GWAS (Yang et al., 2021; Amo and Soriano, 2022; Saini et al., 2022). These phenomena may be ascribed to the material utilized for initial QTL mapping (finally used for MQTL analysis) and GWAS.

Identification of underlying genes in genomic regions of major QTLs is of great interest in breeding programs. More so, MQTLs with narrow and precise physical regions are beneficial in candidate gene mining. According to Kaur et al. (2021), MQTLs with 5-8 initial QTL clusters with moderately high R^2^ values could be considered important for mining candidate genes. The genes present in the 8 MQTLs regions were analyzed using rice databases (http://www.ricedata.cn, http://shigen.nig.ac/jp/rice/oryzabase/, and http://rice.uga.edu.cgi-bin/gbrowse/rice). We identified 2,030 genes and as many as 327 genes were found to encode proteins (sugar transporters, PPR repeat-containing protein-like genes, cytochrome P450, zinc fingers, F-box genes, PP2C family, MADs-Box domain-containing protein, AP2 domain-containing protein, and cytokinase oxidase genes) associated with yield (Wu et al., 2016; Chen et al., 2020; Jiang et al., 2022).

Several genes of unknown functions were also found in stable MQTLs intervals; further study is necessary to explore the functional role of those genes in regulating grain yield. A bioinformatics pipeline implementing the genomic sequences of stable MQTLs was used to identify potential candidate genes. These pipelines involved three steps, thus retrieving candidate genes from rice genome project annotation database, visualizing the molecular function of candidate genes by GO enrichment analysis, and implying candidate genes in rice yield-related tissues based on their expression pattern. Consequently, 52 genes were considered potential candidate genes (**Table 3**). Most of the identified candidate genes are associated with the terms of binding, catalytic activity, cell, cell part, cellular, and metabolic process. These terms have functions related to yield (Mao et al., 2010; Li and Li, 2014; Elattar et al., 2021). In the stable MQTL regions, most of the candidate genes encode zinc finger family protein, ubiquitin ligase domain-containing protein, auxin-responsive gene family, AP2 domain-containing protein, cytochrome P450, receptor-like kinase protein, and MADs-box family protein. Furthermore, zinc fingers have been reported to play a vital role in plant growth, development, and response to abiotic stresses (Li et al., 2013). For instance, a zinc finger protein DST regulates the expression of *OsCKX2* and increases cytokinin level in reproductive shoot apical meristem, which in turn elevates meristem activity, panicle branching, and increased grain number (Li et al., 2013). Similarly, DHHC-zinc finger protein gene has been reported to regulate plant architecture and grain yield (Zhou et al., 2017). AP2 domain-containing proteins have been reported to play essential roles in regulating plant growth and development. Overexpression of *OsAP2-39* decreased biomass and grain yield in transgenic lines of rice (Yaish et al., 2010). Overexpression of OsIAA10 (auxin-responsive gene in MQTL2.6) was reported to increase rice susceptibility to rice dwarf virus (Jin et al., 2016; Qin et al., 2020). One reported gene, *OsBIHD1* (encodes homeodomain protein, putative, expressed) was found in MQTL3.5. Overexpression of *OsBIHD1* revealed an increased salt sensitivity and oxidative stress in rice (Luo et al., 2005). In addition, a clone gene *Oshox22* (encodes homeobox associated leucine zipper, putative, expressed) was identified in MQTL4.2. Overexpression of *Oshox22* increased ABA sensitivity and reduced salt drought and stress tolerance (Zhang et al., 2012).

In MQTL5.2 lies two reported genes, *RSR1* and *OsAPIP6*. The overexpression of *RSR1* resulted in the change of amylopectin and starch gelatinization (Fu and Xue, 2010). *OsAPIP6* interacted with AvrPiz-t *in vitro* and reduced flg22-induced ROS generation, suppressed defense gene expression, and increased rice plant susceptibility to M. *Oryza* (Park et al., 2012). We also found two reported genes, *OsPUB44* (encodes U-box domain-containing protein, expressed) and *OsMADS4* (encodes MADS-box family gene with MIKCc type-box) in MQTL5.5. *OsPUB44* positively control immune responses of rice plant to bacterial blight (Ishikawa et al., 2014). In another study, U-box domain family protein has been reported to play a vital role in rice pollen development (Chen et al., 2019). A mutant of *OsPUB73* (U-box domain protein) showed low pollen fertility suggesting that *OsPUB73* was responsible for pollen exine or tapetal development and led to pollen partial sterility (Chen et al., 2019). The genes, *OsMADS4* interacted with rice AP3 orthologs SPW1 and facilitate stamen and lodicule development (Yao et al., 2008). In previous studies, MADs box genes have been implicated to modulate stamen development in rice (Yamaguchi et al., 2006; Hu et al., 2011; Yan et al., 2015; Wei et al., 2019), arabidopsis (Ito et al., 2004; Ito et al., 2007), and maize (Schreiber et al., 2004). Two genes, *CYP94C2b* (cytochrome P450, putative, expressed) and *OsARF24* (auxin response factor, putative, expressed) underlie MQTL 12.2 have been reported to enhance salt tolerance in rice (Li et al., 2014) and control cell growth (Kurotani et al., 2015).

## Conclusion

In the present study, we integrated QTLs conferring spikelet fertility, panicle number per plant, thousand-grain weight, and number of grains per panicle which led to the identification of 62 MQTLs. 16 of these MQTLs were successfully verified with GWAS studies on the studied traits. 10 MQTLs that harbored at least six of the initial QTLs were selected as the most stable and robust chromosome regions to mine CGs and positional cloning that could be useful for breeding programs. Based on the bioinformatics pipelines, 52 potential CGs were identified to be associated with grain yield. The high expression of these genes was evidenced in yield-related tissues and maybe grain yield regulatory genes. This study provides a useful framework for genetic dissection of grain yield under unstressed conditions *via* positional cloning, fine mapping, and CGs validation by gene editing technique. The remaining novel CGs detected in this study could be validated or functionally characterized *via* gene editing, overexpression, knockout techniques, and candidate gene-based association mapping to ascertain their roles in regulating grain yield in future studies.

## Data availability statement

The original contributions presented in the study are included in the article/**Supplementary Material**. Further inquiries can be directed to the corresponding author.

## Author contributions

XT, NEO, and KDA conceptualized and design the study. XT supervised the study. KDA, AA, and NEO curated data and perform the analysis. KDA wrote the manuscript. SFB, SA, and AA revised and edited the manuscript. All authors have read and approved the final version of the manuscript.

## Funding

This study was supported by The Key R&D Project in Hubei Province, China (2020BBB060).

## Conflict of interest

The authors declare that the research was conducted in the absence of any commercial or financial relationships that could be construed as a potential conflict of interest.

## Publisher’s note

All claims expressed in this article are solely those of the authors and do not necessarily represent those of their affiliated organizations, or those of the publisher, the editors and the reviewers. Any product that may be evaluated in this article, or claim that may be made by its manufacturer, is not guaranteed or endorsed by the publisher.
