# Supplementary figures and images for: A meta-quantitative trait loci analysis identified consensus genomic regions and candidate genes associated with grain yield in rice
Source: Front Plant Sci. 2022 Nov 16;13:1035851. doi: 10.3389/fpls.2022.1035851 (PMC9709451; doi:10.3389/fpls.2022.1035851)

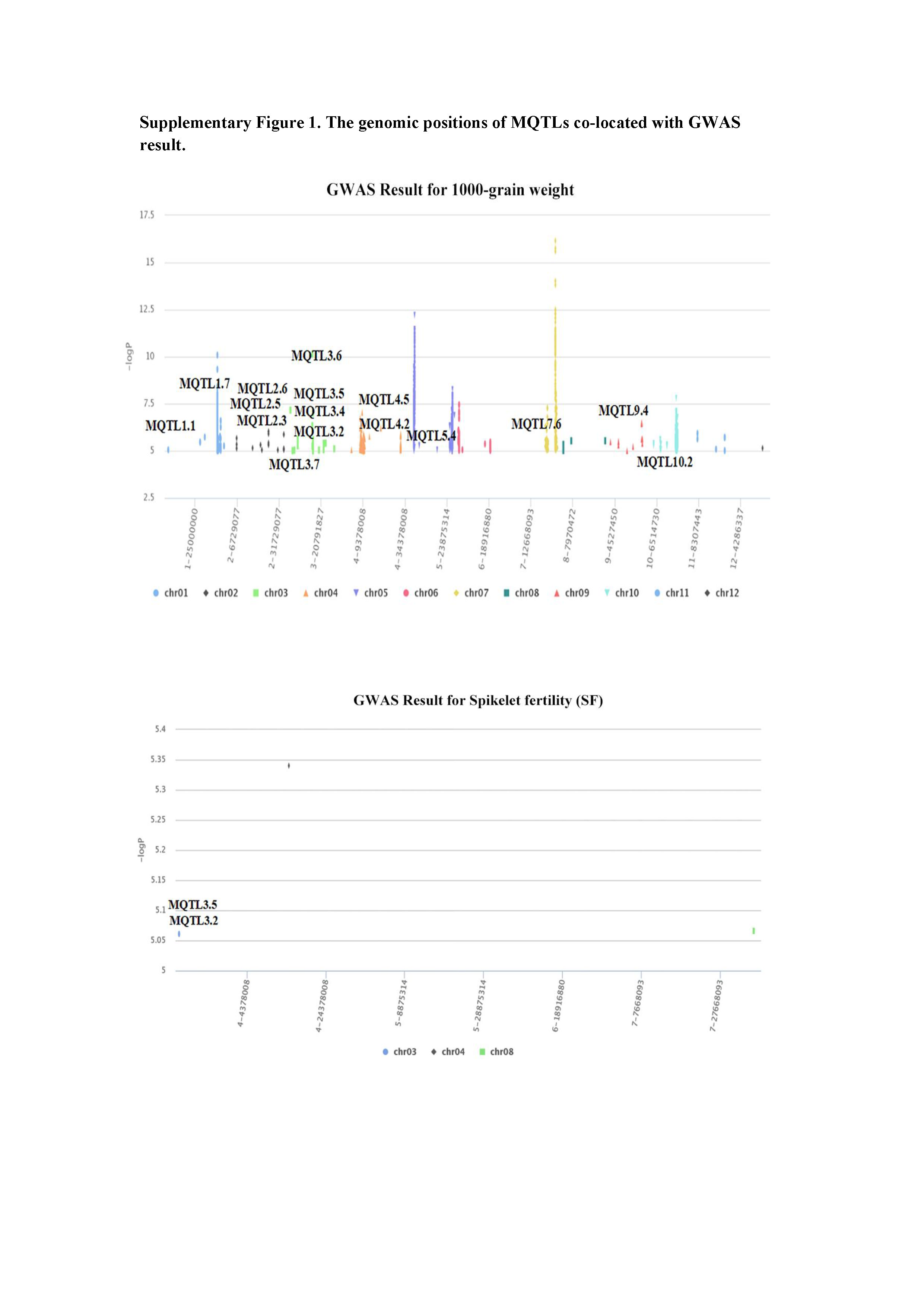

Supplement: Supplementary file 1 [file Image_1.tif]
